# Approach for determining micro-strength parameters of rock based on particle flow code

**DOI:** 10.1038/s41598-024-61410-x

**Published:** 2024-05-16

**Authors:** Luli Miao, Xinrong Liu, Yan Fu

**Affiliations:** 1 School of Civil Engineering, Chongqing University, Chongqing, 400045 China; 2 School of Construction Management and Real Estate, Chongqing University, Chongqing, 400045 China; 3 Center for Construction Economics and Management, Chongqing University, Chongqing, 400045 China

**Keywords:** Particle flow code (PFC), Plackett–Burman (PB) design, Brazil disk split test, Micro parameters, Petrology, Civil engineering, Natural hazards

## Abstract

Discrete Element Method (DEM) has been successfully utilized to model rock behavior based on particle flow code (PFC), which is extensively employed in solving various problems related to rock engineering and geomechanics. Therefore, a convenient method for selecting appropriate microparameters of PFC for model generation is necessary. The present study aims to develop a novel approach that calculates proper micro-strength parameters for the contact bond model (CBM). Firstly, based on Plackett–Burman (PB) design, qualitative research is conducted and it is found that the main factors that influence the Brazilian tensile strength is microscopic tensile strength. We analyzed the stress conditions of a Brazilian disc’s vertical diameter using both continuum models and DEM. From this analysis, we establish a theoretical relationship between rock tensile strength and micro-strength parameters. Subsequently, a large number of numerical Brazilian tests were conducted to obtain the statistical relationship between the geometric parameters of balls, micro-strength parameters and the Brazilian tension strength. The results of the numerical simulation were then used to refine the theoretical equation mentioned above, resulting in a modified equation for rock tensile strength and micro-strength parameters. Finally, after verification, we confirm the feasibility of the method in this paper.

## Introduction

Particle flow code (PFC), as a discrete element method (DEM) based on the micromechanical properties of particulate media, was widely used in the simulation of loose media such as sands in the early days. It was not until the proposal of a Bonded Particle Model (BPM) for rock by Potyondy and Cundall^1^ and the successful explanation of the physical mechanism for rock pressure-induced tensile fracturing that the PFC became a powerful tool for analyzing the complex mechanical behavior of rocks.

Different from the macro-mechanical parameters of material, the micro-mechanical parameters of PFC cannot be obtained directly through laboratory test, which are obtained indirectly by a method called “trial and error”. This method often consumes considerable time and effort of the researchers. In addition, when PFC is used to simulate the rock mechanical behavior, the mechanical response of rock is influenced jointly by the geometric parameters of particles and the micro-strength parameters. Therefore, it appears very important to study the correlation between macro- and micro-parameters, as well as the method of setting PFC micro-parameter values.

Scholars at home and abroad have conducted a series of studies on the correlation and value range of macro- and micro-parameters for rock particle flow models. Potyondy and Cundall^1^ indirectly suggested that the macroscopic tensile strength was correlated with the normal bond strength and particle geometric parameters by studying the fracture toughness. Huang^2^ studied the qualitative relationship of micro-parameters with elastic modulus, Poisson’s ratio, uniaxial compressive strength and fracture toughness in CBM by means of dimensional analysis combined with uniaxial, biaxial simulations. Yang et al.^3^ obtained the dimensionless quantitative relation of uniaxial compressive strength, Young’s modulus and Poisson’s ratio to micro-parameters through statistic analysis by simulating a large number of uniaxial compression tests. Yoon et al.^4^ performed sensitivity analysis on the macro-parameters (uniaxial compressive strength, Young’s modulus, Poisson’s ratio) and micro-parameters in the uniaxial compression test using Plackett–Burman (PB) design. For each macroscopic response, the two largest micro-influence factors were selected, and the response surface and nonlinear relationship of macro- and micro-parameters were obtained by central composite design (CCD). Finally, the parameters were optimized to obtain micro-parameters that allow good simulation of specimens. Although J. Yoon et al. derived a fairly complete CBM micro-parameter selection method, its process is cumbersome and thus is hardly applicable. Wang et al.^5–7^ proposed the improved particle swarm optimization (PSO) calibration method, the salp swarm optimization (SSO) algorithm, and the improved simulated annealing algorithm to obtain the microparameters of the DEM model. Wu et al.^8^ proposes a method of inverse performance of the regression equations of the macroscopic parameters by the gray absolute correlation combined with regression analysis to calibrate the microscopic parameters of the siltstone. In the above studies, Potyondy and Cundall^1^ and Huang^2^ completed the qualitative analysis of macro- and micro-parameters, which cannot be directly applied because of the lack of definite quantitative relations; Yang et al.^3^ and Yoon et al.^4^ obtained the quantitative relations for macro- and micro-parameters based on substantial experimental data, which though had insufficient theoretical derivation basis, thus limiting their scope of application by the test data samples. Moreover, the focus of studies was the macro- and micro-parameters in uniaxial compression test, while the macroscopic tensile strength was seldom analyzed.

In this paper, a numerical model of Brazilian disc is built by taking the Contact Bond Model (CBM) for rock as the research object. The responses of macro-mechanical parameters of rock are studied by Design of Experiments (DOE), and the main micro-mechanical parameter influencing Brazilian strength is identified. Then, the semi-analytical solution of discrete particle model is established based on the Brazilian disc analytical solution of continuum model to obtain a scale coefficient *K*’, based on which the theoretical relationship between Brazilian strength and micro-strength parameters is established. Finally, solving of scale coefficient *K*’ is completed through 350 runs of PFC numerical simulations.

## Sensitivity analysis of microparameters by DOE

### Bonded particle model and DOE

Currently, there are multiple contact models available in PFC^2D^, among which the contact models suitable for simulating rock-like materials include Linear Contact Bond Model (CBM), Linear Parallel Bond Model (PBM), Smooth-Joint Contact Model (SJM), and Flat-Joint Contact Model (FJM). Although CBM and PBM, as early developed contact bonding models, have some drawbacks when simulating rock materials, compared to other models, CBM and PBM involves fewer micro-parameters. As shown in Table 1, the contact position of CBM only involves five micro-parameters while PBM involves seven. Excessive micro-parameters would greatly increase the difficulty of studying the macro–micro parameter theory relationship. Therefore, we will first explore the theoretical and statistical relationship between macro–micro parameters of bonding materials using CBM as an example.

**Table 1:** Micro-parameters of different contact constitutive models in PFC^2D^.

Type of material		Microparameters of grain	Microparameters of stiffness		Microparameters of strength	
			Grains	Cement	Grains	Cement
Unbonded material	Linear model	*ρ*, *R*_min_, *R*_max_	*E*_c_, *k*_n_/*k*_s_	–	μ	–
Bonding material	Contact bond model	*ρ*, *R*_min_, *R*_max_	*E*_c_, *k*_n_/*k*_s_	–	μ	*σ*_cn_, *τ*_cs_
	Parallel bond model	*ρ*, *R*_min_, *R*_max_, $\overline{\lambda }$	*E*_c_, *k*_n_/*k*_s_	$\overline{E} _{\text{c}}$, $\overline{k}_{\text{n}}/\overline{k}_{\text{s}}$	μ	$\overline{\sigma }_{{{\text{cn}}}}$, $\overline{\tau }_{{{\text{cs}}}}$

The macro-responses of micro-parameters can be completed by DOE. As mentioned earlier, CBM has seven independent micro-parameters. If full factorial design is adopted, each parameter will be subjected to a 2-level test, so a total of 2^7^ = 128 numerical tests will be required. To reduce the number of numerical tests while obtaining the effect of micro-parameters on the macroscopic tensile strength, the PB design method^9^ with fewer tests can be used. For PB design consisting of seven independent parameters, only 12 numerical tests are required.

### Range of microparameters selection and correction

The seven micro-parameters of CBM can be selected according to the following approach: The particle geometric parameters are expressed by minimum ball radius *R*_min_ and radius ratio *R*_max_/*R*_min_; and the micromechanical parameters are expressed by contact modulus *E*_c_, stiffness ratio of normal to tangential *k*_n_/*k*_s_, normal bond strength *σ*_cn_, strength ratio of normal to tangential *σ*_cn_/*τ*_cn_ and particle friction coefficient *μ*. The ranges of micro-parameters are selected based on the previous studies^1,3,4,10–17^, which are detailed in Table 2. According to the statistical results in Table 2, the upper and lower limits of the micro-parameters can be derived, which correspond to the minus level and plus level of factors, respectively, see Table 3. The PB test run sequences are detailed in Table 4.

**Table 2:** Sumarry of micro-parameters for PFC^2D^ model.

Reference	Simulation object	Microparameters									Macro response			
		*R*_max_/*R*_min_	*R*_min_(mm)	L/R	*E*_c_(GPa)	*k*_n_/*k*_s_	*σ*_cn_(MPa)	*τ*_cs_(MPa)	*σ*_cn_/*τ*_cs_	μ	*σ*_t_(MPa)	*σ*_ci_(MPa)	*E*(GPa)	ν
Potyondy and Cundall^1^	Lac du Bonnet granite	1.66	–	88.1 mean radius	62	2.5	157 ± 36	157 ± 36	1	0.5	44.7 ± 3.3	71.8 ± 21.8	70.9 ± 0.9	0.237 ± 0.011
Yang^3^	Rock material	1.2–6.2	0.135	20–260 mean radius	38	0.8–2.7	46	92	0.25–3.5	0.1–0.8	–	75.9–165.6	34.2–68.4	0.09–0.3
Yoon^4^	Rock material	1.66	0.24–0.48	78.3–156.6	40–100	1.0–4.0	50–200	50–200	–	0.25–0.75	9.6–51.6	40–170	20–50	0.19–0.25
Fu^10^	Sandstone	4	0.3–0.5	40–66.7	4–16	1.5–6	1.48–80	8.16–87.5	–	0.01–10	–	3.27–130.62	2.52–10.69	0.166–0.338
			0.4	50	11.5	3.5	18.4	89.7	0.205	1.95	–	33.13	6.76	–
Zhao^17^	Rock material	1.5–5		20–250	10–32	0.2–6	–	–	0.2–3.5	0.1–1.0	–	20–170	(0.88–2.24)Ec	0.1–0.45
	Quartzite	1.3	0.075	695	40	1.2	69 ± 15	138 ± 30	0.5	0.25	–	–	–	–
	Siltstone	1.3	0.075	695	33	1.5	40	100	0.8	0.75	–	188	38.66–40.22	0.22–0.28
Li ^14^	Granite				3.6	2.1	45.12 ± 10	45.12 ± 10	1	0.5				
N. Cho^11^	Lac du Bonnet granite	1.5	0.20		20	2.5	25 ± 3.5		0.05	0.1				
Huang^13^	Rock-like material	1.6	0.15–0.45	0.195–0.585	2.3	2.0	11 ± 1.5	15 ± 2.5		0.45				
Hadi Haeri^12^	Rock material	1.56	0.27		40	1.7	25 ± 2	25 ± 2		0.4				
Yang^15^	Rock material	1.66	0.3		30	3	70 ± 14	80 ± 16		0.65				
Yang^16^	Rock material				2.3	2.0	22 ± 1.5	15 ± 2.5		0.45

**Table 3:** Microparameters needed for generation of contact-bonded particle model.

Microparameters(Abbreviation &unit)	Uncoded values of each level			Transformation from coded to uncoded
*X*_1_ ~ *X*_7_	− 1	0	+1	(coded = − 1 ~ + 1)
*X*_1_: Minimum ball radius (*R*_min_) (mm)	0.135	0.2675	0.4	Uncoded = 0.1325 × coded + 0.2675
*X*_2_: Radius ratio (*R*_max_/*R*_min_) (dimensionless)	1.2	3.7	6.2	Uncoded = 2.5 × coded + 3.7
*X*_3_: Ball-to-ball Contact Modulus (*E*_c_) (GPa)	4	52	100	Uncoded = 48 × coded + 52
*X*_4_: Stiffness ratio (*k*_n_/*k*_s_) (dimensionless)	0.2	3.1	6	Uncoded = 2.9 × coded + 3.1
*X*_5_: Contact normal bond strength *σ*_cn_(Mpa)	1.48	100.74	200	Uncoded = 99.26 × coded + 100.74
*X*_6_: Strength ratio (*σ*_cn_/*τ*_cs_) (dimensionless)	0.2	0.6	1	Uncoded = 0.4 × coded + 0.6
*X*_7_: Ball friction coefficient (*μ*) (dimensionless)	0.01	5.005	10	Uncoded = 4.995 × coded + 5.005

**Table 4:** Complete design matrix for Plackett–Burman design.

NO	*X*_1_: *R*_min_	*X*_2_: *R*_max_/*R*_min_	*X*_3_: *E*_c_	*X*_4_: *k*_n_/*k*_s_	*X*_5_: *σ*_cn_	*X*_6_: *σ*_cn_/*τ*_cs_	*X*_7_: *μ*	Result
1	− 1	+1	+1	− 1	+1	− 1	− 1	Not available
2	− 1	+1	+1	+1	− 1	+1	+1	Not available
3	+1	− 1	+1	− 1	− 1	− 1	+1	Available
4	+1	+1	+1	− 1	+1	+1	− 1	Not available
5	+1	− 1	+1	+1	− 1	+1	− 1	Available
6	+1	− 1	− 1	− 1	+1	+1	+1	Available
7	+1	+1	− 1	+1	− 1	− 1	− 1	Available
8	− 1	+1	− 1	− 1	− 1	+1	+1	Available
9	− 1	− 1	− 1	+1	+1	+1	− 1	Available
10	+1	+1	− 1	+1	+1	− 1	+1	Available
11	− 1	− 1	+1	+1	+1	− 1	+1	Available
12	− 1	− 1	− 1	− 1	− 1	− 1	− 1	Available

In Table 2: *σ*_t_ is the Brazilian tensile strength, *σ*_ci_ is the uniaxial compressive strength, *E* is the Young’s modulus, *ν* is the Poisson’s ratio; L is the minimum characteristic length of specimen (diameter of specimen for cylinders, discs; and height of specimen for cuboid); and *R* is the median between maximum and minimum ball radius. In the annotated L/R parameter, *R* is the average particle size.

According to ISRM^18^, the diameter of Brazilian disc is set to 50 mm. Calculation of PB design is performed according to the combinations in Table 4. The trial calculation reveals abnormal calculation results for some combinations. These combinations are characterized by: excessively large radius ratio, excessively large or small ratio of contact modulus to normal bond strength, as well as excessively small stiffness ratio. Modification is required for these micro-parameters.

- Radius ratio modification

Huang^2^ used *L*/*R* to describe the discrete degree of Discrete Element Model, where *L* represented the minimum characteristic length of the model, and *R* represented the median particle size. Some researchers also used *L*/*R* to describe the relative size of specimen constituting particles for particle size analysis under different specimen dimensions. According to the value range of *L*/*R* in Table 2, this paper preliminarily determines *R*_min_ under uniaxial test specimen dimensions of 50 mm × 100 mm. Meanwhile, the lower limit of minimum specimen particle size is constrained by computer configuration, while excessively large upper limit disables equilibrium state during the specimen generation. *R*_min_ is finalized as 0.135–0.4 mm.

**Figure 1:**
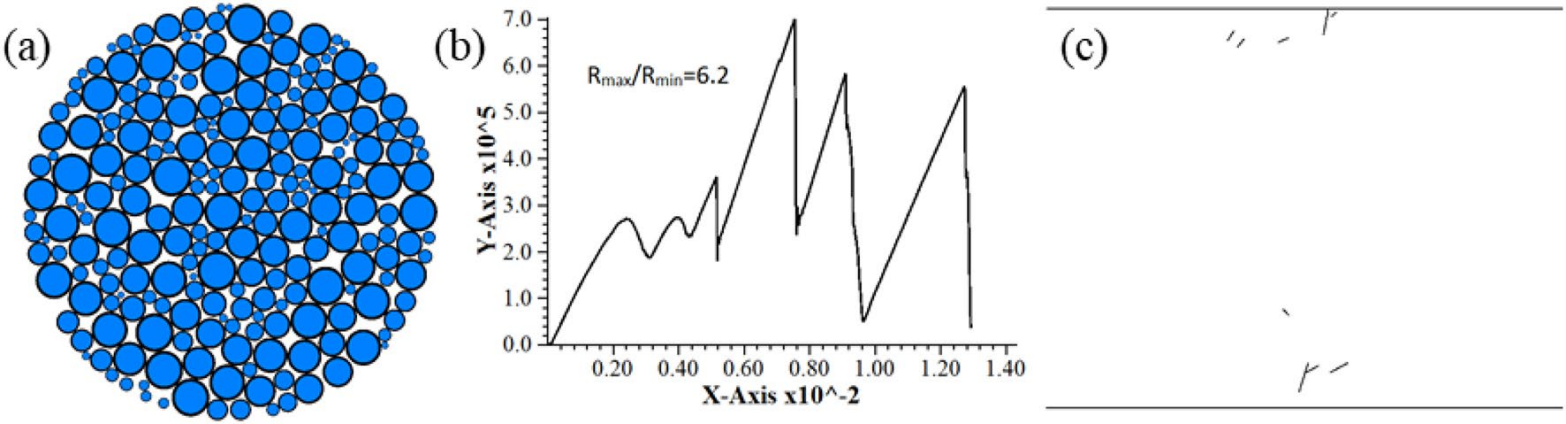
Simulation results of not available model before correction. (**a**) Model (**b**) Load–displacement curve (**c**) Post-failure fracture distribution.

**Figure 2:**
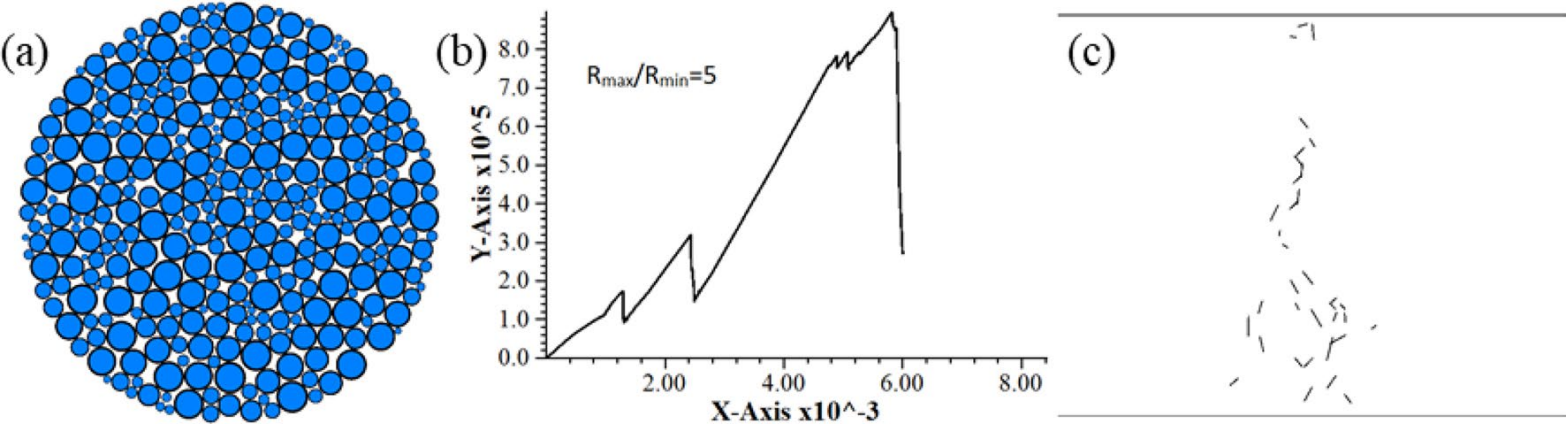
Simulation results after the correction of radius ratio. (**a**) Model (**b**) Load–displacement curve (**c**) Post-failure fracture distribution.

During the tests, the disc rotated when the plus level of radius ratio was 6.2. Figure 1a shows the particle flow model. The shape of specimen’s force–displacement curve and the fracture distribution did not match the results of laboratory rock test, as shown in Fig. 1b and c. The specimen could not get the correct tensile strength. Figure 2a presents the particle flow model after *R*_max_/*R*_min_ adjustment to 1.2–5 and modification of radius ratio, while Fig. 2b, c illustrate the load–displacement curve and the fracture distribution after specimen failure. It should be noted that the above-mentioned determination of radius ratio was done on the premise of corresponding micro-parameters of this study.

b. Modification of contact modulus and normal bond strength

**Figure 3:**
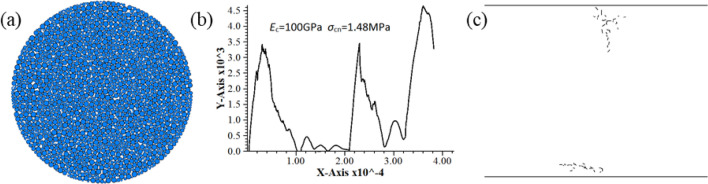
Simulation results of not available model before correction. (**a**) Model (**b**) Load–displacement curve (**c**) Post-failure fracture distribution.

**Figure 4:**
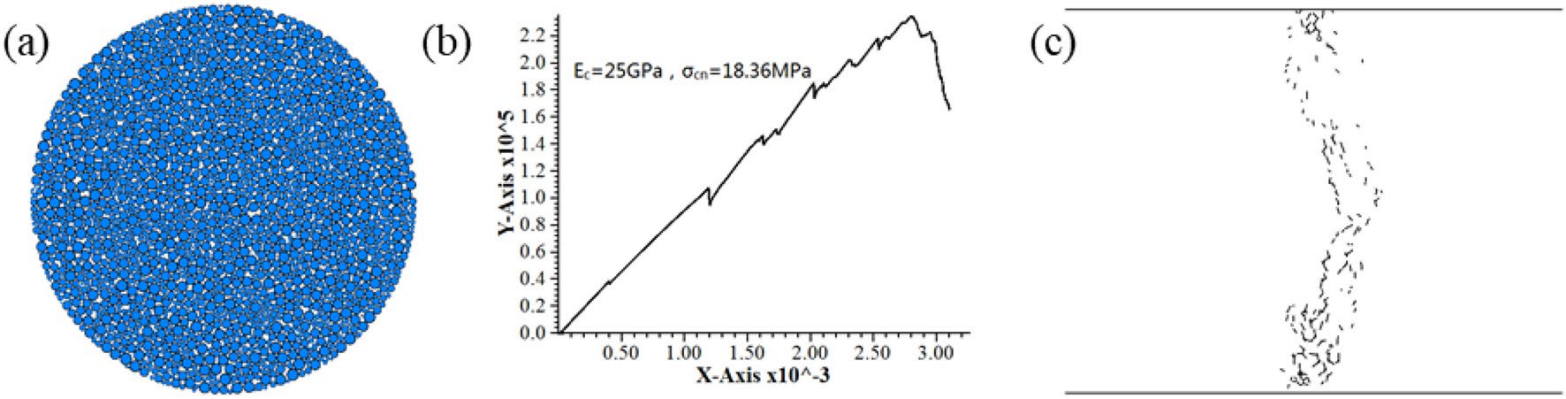
Simulation results after the correction of contact modulus and contact normal bond strength. (**a**) Model (**b**) Load–displacement curve (**c**) Post-failure fracture distribution.

According to Table 2, it is commonly observed that scholars typically consider the normal/tangential strength ratio within the range of 0.2–3.5 when investigating the correlation between macro and micro parameters. Moreover, based on calibrated parameters, it has been determined that materials exhibiting rock mechanical characteristics are more readily attainable with a normal/tangential strength ratio below 1. Consequently, a range of 0.2–1 is selected for the normal/tangential strength ratio. It can be seen from the micro-parameter values selected by Fu^10^, Yoon^4^ and Zhao^17^ that the contact modulus and normal bond strength of simulated sandstone were both smaller than the simulated granite. If the minus level and plus level of contact modulus and normal bond strength are taken as the minimum and maximum values in Table 2, respectively, small contact modulus and large normal bond strength or large contact modulus and small normal bond strength will appear during PB test. As shown in Fig. 3b and c, irrational combination of these two parameters results in abnormal test curve and fracture development. So, the test values of contact modulus and normal bond strength should be carefully selected. Calculation found that the ratio of contact modulus to normal bond strength for rock simulation was within a range of (0.14–0.83) × 10^3^ in Table 2, while that for unreasonable specimen was up to 67.5 × 10^3^ in Fig. 3. The above statistics show that the ratio of contact modulus to normal bond strength must be controlled within a certain range, in order to ensure that the parameter combination can reasonably simulate the rock material. The contact modulus and normal bond strength in this study are adjusted based on an interval (0.14–0.83) × 10^3^ to obtain contact modulus of 11–25 GPa, and normal bond strength of 18.36–52.12 MPa. The ratio of the two is (0.211–1.360) × 10^3^, which basically meets the test requirements. Figure 4 presents the particle flow model of specimen after adjustment, force–displacement curve and post-peak fracture morphology, which basically agree with the results of laboratory rock test.

c. Modification of normal/tangential stiffness ratio.

**Figure 5:**
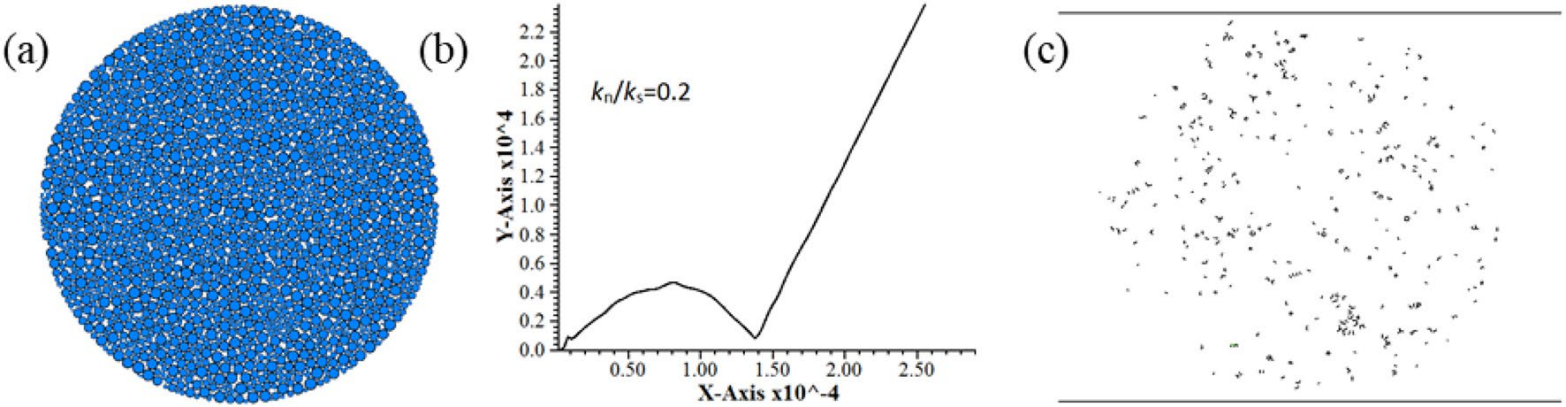
Simulation results of not available model before correction. (**a**) Model (**b**) Load–displacement curve (**c**) Post-failure fracture distribution.

**Figure 6:**
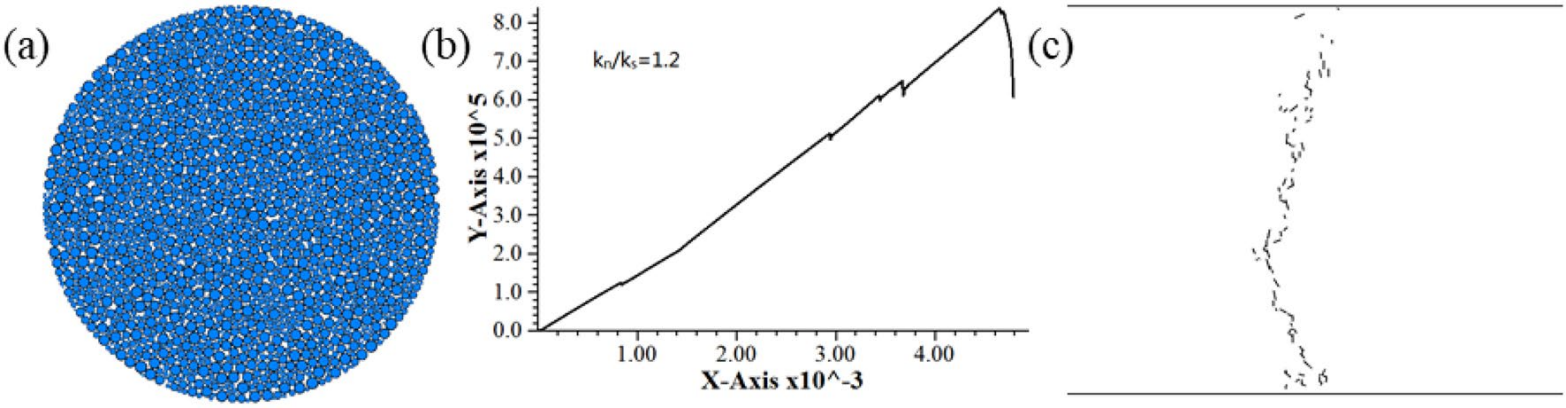
Simulation results after the correction of stiffness ratio. (**a**) Model (**b**) Load–displacement curve (**c**) Post-failure fracture distribution.

In the course of test, abnormal fracture development of specimen occurred as a result of excessively small normal/tangential stiffness ratio (as shown in Fig. 5). In Fig. 5c, all the fractures are shear fracture; the fractures are distributed over the entire disc rather than extending along the vertical diametral direction; all the fractures are distributed around the small particles. The normal bond strength and tangential bonding strength of the specimen are both the maximum values in the PB test, but the fractures are produced at small load values, which are obviously inconsistent with the results of laboratory rock test as well. Normal/tangential stiffness ratios in Table 2 for the calibration rock model are all greater than 1. Therefore, the minus level of normal/tangential stiffness ratio is modified to 1.2, then its range of study should be 1.2 and 6. The experimental results after stiffness ratio adjustment are shown in Fig. 6, where the force–displacement curve and fracture expansion are both consistent with the results of laboratory rock test.

### Sensitivity analysis of microparameters using PB design

After several tests and modifications, a set of PB test parameter values that can reasonably describe the mechanical properties of rock is finalized (see Table 5). Based on the PB design method, this paper designs a 7-factor 2-level PB matrix (see Table 5) for a total of 12 runs of numerical experiments, the specific plan is shown in Table 6. The specimen is a disc with a diameter of 50 mm. The numerical results are shown in Table 6, where the tensile strength is within a range of 1.74–18.96 MPa.

**Table 5:** Microparameters needed for generation of contact-bonded particle model.

Microparameters(Abbreviation &unit)	Uncoded values of each level			Transformation from coded to uncoded
*X*_1_ ~ *X*_7_	− 1	0	+ 1	(coded = -1 ~ + 1)
*X*_1_: Minimum ball radius (*R*_min_) (mm)	0.135	0.2675	0.4	Uncoded = 0.1325 × coded + 0.2675
*X*_2_: Radius ratio (*R*_max_/*R*_min_) (dimensionless)	1.2	3.1	5	Uncoded = 1.9 × coded + 3.1
*X*_3_: Ball-to-ball contact modulus (*E*_c_) (GPa)	11	18	25	Uncoded = 9 × coded + 18
*X*_4_: Stiffness ratio (*k*_n_/*k*_s_) (dimensionless)	1.2	3.6	6	Uncoded = 2.4 × coded + 3.6
*X*_5_: Contact normal bond strength σ_cn_(Mpa)	18.36	35.24	52.12	Uncoded = 16.88 × coded + 35.24
*X*_6_: Strength ratio (*σ*_cn_/*τ*_cs_) (dimensionless)	0.2	0.6	1	Uncoded = 0.4 × coded + 0.6
*X*_7_: Ball friction coefficient (*μ*) (dimensionless)	0.01	5.005	10	Uncoded = 4.995 × coded + 5.005

**Table 6:** Complete design matrix for Plackett–Burman design and test results.

NO	*X*_1_: R_min_	*X*_2_: R_max_/R_min_	*X*_3_: E_c_	*X*_4_: k_n_/k_s_	*X*_5_: σ_cn_	*X*_6_: σ_cn_/τ_cs_	*X*_7_: μ	*Y*_1:_*σ*_t_(MPa)
1	− 1	+1	++1	− 1	+1	− 1	− 1	13.74
2	− 1	+1	+1	+1	− 1	+1	+1	3.85
3	+1	− 1	+1	− 1	− 1	− 1	+1	3.57
4	+1	+1	+1	− 1	+1	+1	− 1	11.42
5	+1	− 1	+1	+1	− 1	+1	− 1	1.74
6	+1	− 1	− 1	− 1	+1	+1	+1	13.85
7	+1	+1	− 1	+1	− 1	− 1	− 1	4.61
8	− 1	+1	− 1	− 1	− 1	+1	+1	3.55
9	− 1	− 1	− 1	+1	+1	+1	− 1	10.31
10	+1	+1	− 1	+1	+1	− 1	+1	10.78
11	− 1	− 1	+1	+1	+1	− 1	+1	18.96
12	− 1	− 1	− 1	− 1	− 1	− 1	− 1	8.52

**Figure 7:**
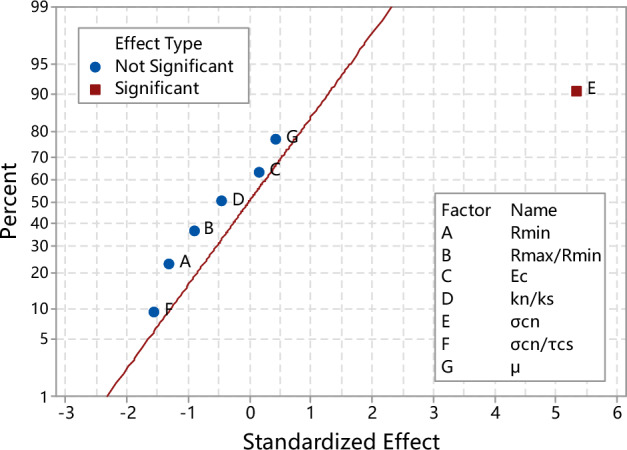
Normal probability plot of the standardized effects for Brazilian tensile strength.

Factor analysis is performed on the macroscopic response (tensile strength) at a 95% CI, i.e. a = 0.05, to make the standardized effect normal plot of tensile strength as shown in Fig. 7. In the figure, the parameter marked with square is precisely the micro-parameter with significant influence. Clearly, normal bond strength has a significant influence on the Brazilian tensile strength.

The PB experiment qualitatively demonstrates the significant relationship between macroscopic and microscopic tensile strengths. The results of many scholars ^1,19,20^ also indirectly indicated the significant relationship between macroscopic and microscopic tensile strengths from the statistical and theoretical perspectives.

## Mechanical analysis of Brazilian disk

### Analytical solution of Brazilian disk based on continuum model

**Figure 8:**
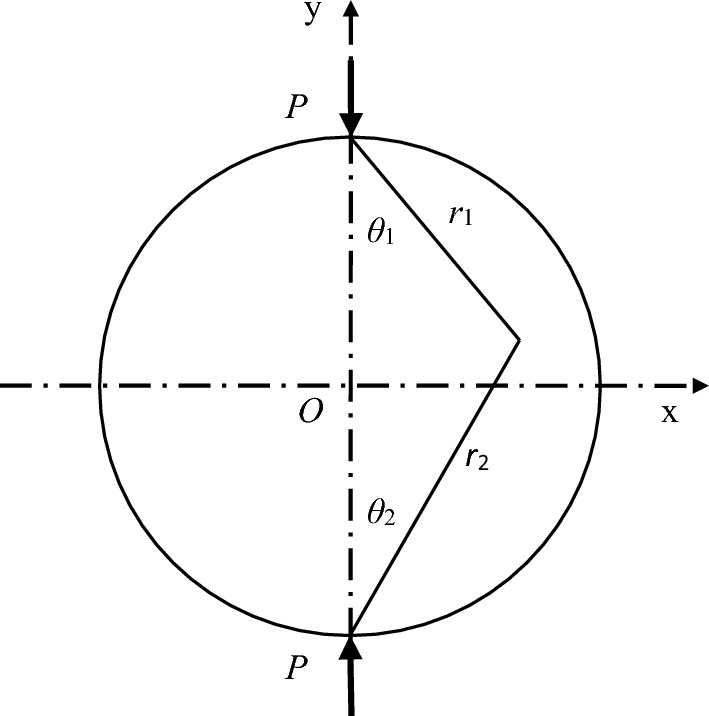
The stress diagram of Brazilian specimen^17^.

The stress state of Brazilian disc splitting test is shown in Fig. 8.

According to the elastic mechanical analytical solution to the plane stress problem^21^, the stress state of any point T (x, y) within the disk (with a radius *R*) can be derived:

1 $$\sigma_{{\text{x}}} = \frac{2P}{{\pi L}}\left( {\frac{{\sin^{2} \theta_{1} \cos \theta_{1} }}{{r_{1} }} + \frac{{\sin^{2} \theta_{2} \cos \theta_{2} }}{{r_{2} }}} \right) - \frac{2P}{{\pi DL}}$$

2 $$\sigma_{{\text{y}}} = \frac{2P}{{\pi L}}\left( {\frac{{\cos^{3} \theta_{1} }}{{r_{1} }} + \frac{{\cos^{3} \theta_{2} }}{{r_{{2}} }}} \right) - \frac{2P}{{\pi DL}}$$

3 $$\tau_{{\text{x}}} = \frac{2P}{{\pi L}}\left( {\frac{{\sin \theta_{1} \cos^{2} \theta_{1} }}{{r_{1} }} - \frac{{\sin \theta_{2} \cos^{2} \theta_{2} }}{{r_{2} }}} \right)$$

where* P* is the loading force, *L* is the specimen thickness, and D is the specimen diameter.

Under the action of line load *P*, there is *θ*_1_ = *θ*_2_ = 0° on the vertical diameter of specimen. The horizontal tensile stress in the plane along the vertical diameter of specimen can be derived according to Eq. (3):

4 $$\sigma_{{\text{x}}} = - \frac{2P}{{\pi DL}}$$

where the minus sign indicates that all forces are tensile.

The horizontal stress in the plane along the vertical diameter of disk is fixed, so the horizontal resultant in the plane is

5 $$F_{{\text{x}}} = \sigma_{{\text{x}}} \times D = - \frac{2P}{{\pi L}}$$

### Mechanical analysis of Brazilian disk based on particle model

**Figure 9:**
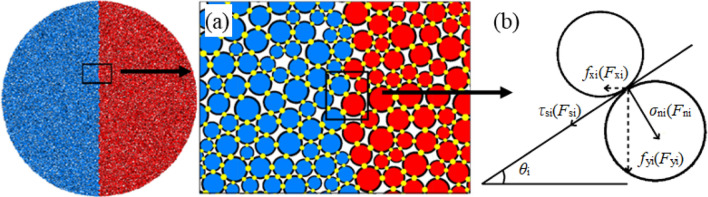
Diagram of calculation model (**a**) Caculating model of PFC^2D^ (**b**) Stress diagram of contact point.

In this paper, PFC^2D^ CBM is adopted, where the particles are point-contacted with each other (as shown in Fig. 9a). The contact points only transmit forces, and the normal stress *σ*_n_ and tangential stress *τ*_s_ at the contact points are transformed into the normal force *F*_n_ and tangential force *F*_s_ as shown in Eqs. (6) and (7).

6 $$F_{{\text{n}}} = \sigma_{{\text{n}}} A$$

7 $$F_{{\text{s}}} = \tau_{{\text{s}}} A$$

$$A = 2r{\text{t}}({\text{t}} = 1),\;r = \left\{ {\begin{array}{*{20}l} {\min \left( {R^{\left( 1 \right)} ,R^{\left( 2 \right)} } \right),} \hfill & {\text{ball - ball}} \hfill \\ {R^{\left( 1 \right)} ,} \hfill & {\text{ball - facet}} \hfill \\ \end{array} } \right.$$

where *R*^(1)^ and *R*^(2)^ are the radii of two particles forming the contact.

As shown in Fig. 9b, assuming that the tangential force of a certain contact point forms *θ*_i_° with the horizon; *F*_ni_, *F*_si_, *σ*_ni_ and *τ*_si_ represent the nominal, tangential contact forces and stresses of the contact point, respectively; and *r*_i_ is the smaller radius of two particles forming contact. This paper discusses 2D model, t = 1. Hence, the horizontal force *F*_x_ at the said contact point is as shown in Eq. (8), where the normal force is positive.

8 $$F_{{{\text{xi}}}} = F_{{{\text{si}}}} \cos \theta_{{\text{i}}} - F_{{{\text{ni}}}} \sin \theta_{{\text{i}}} = 2r_{{\text{i}}} t\tau_{{{\text{si}}}} \cos \theta_{{\text{i}}} - 2r_{{\text{i}}} t\sigma_{{{\text{ni}}}} \sin \theta_{{\text{i}}}$$

It can be seen from the fracture development pattern of Brazilian specimen in the loading phase that a large number of tensile fractures were produced in the vertical diameter region at the moment the load reached a peak, afterwards, the load dropped rapidly. In this paper, the horizontal shear force *F*_x_ borne by the fractures formed during such phase is considered the horizontal tension of specimen at the vertical diameter region in the peak load phase, and the vertical resultant *F*_y_ borne by these fractures is considered the external load *P* applied by the test. The contact to be damaged during the peak load phase is named the key contact. Assuming that the number of key contacts in the specimen is *n*, and the tangential force of each key contact forms *θ*_i_° with the horizontal line, then the horizontal resultant of specimen in the vertical diameter direction during the peak load phase is:

9 $$F_{{\text{x}}} = \sum\limits_{i = 1}^{n} {F_{{{\text{xi}}}} } = \sum\limits_{i = 1}^{n} {(2r_{{\text{i}}} t\tau_{{{\text{si}}}} \cos \theta_{{\text{i}}} - 2r_{{\text{i}}} t\sigma_{{{\text{ni}}}} \sin \theta_{{\text{i}}} } ) = \sum\limits_{i = 1}^{n} {\sigma_{{{\text{ni}}}} \left[ {2r_{{\text{i}}} t\left( {\frac{{\tau_{{{\text{si}}}} }}{{\sigma_{{{\text{ni}}}} }}} \right)\cos \theta_{{\text{i}}} - 2r_{{\text{i}}} t\sin \theta_{{\text{i}}} } \right]}$$

### Semi-analytical solution of Brazilian disc based on particle model

It can be seen from simultaneous Eqs. (5), (9) that the failure of Brazilian disc specimen is manifested mainly as separation fractures, so *σ*_ni_ → *σ*_cn_ when *F*_x_ → 2*P*/π*L*.

10 $$\frac{2P}{{\pi L}} = \sigma_{{{\text{cn}}}} \sum\limits_{i = 1}^{n} {\left[ {2r_{{\text{i}}} t\sin \theta_{{\text{i}}} - 2r_{{\text{i}}} t\left( {\frac{{\tau_{{{\text{si}}}} }}{{\sigma_{{{\text{cn}}}} }}} \right)\cos \theta_{{\text{i}}} } \right]}$$

It can be seen from (10) that the Brazilian strength is related to the normal bond strength, the normal/tangential strength ratio and the particle geometric parameter.

Let

11 $$\sum\limits_{i = 1}^{n} {2r_{{\text{i}}} t\sin \theta_{{\text{i}}} } = \sum\limits_{i = 1}^{n} {m_{{\text{i}}} } = m$$

The physical meanings of *m*_i_ and *m* are: the vertical projection length of a key contact and the sum of vertical projection lengths of all key contacts, respectively.

From Eq. (10), we can get

12 $$\sigma_{{\text{t}}} = \frac{2P}{{\pi DL}} = \sigma_{{{\text{cn}}}} \left( {\frac{{m - \sum\limits_{i = 1}^{n} {2r_{{\text{i}}} t\left( {\frac{{\tau_{{{\text{si}}}} }}{{\sigma_{{{\text{cn}}}} }}} \right)\cos \theta_{{\text{i}}} } }}{D}} \right)$$

From Eq. (12), we can get

13 $$K = \frac{{m - \sum\limits_{i = 1}^{n} {2r_{{\text{i}}} t\left( {\frac{{\tau_{{{\text{si}}}} }}{{\sigma_{{{\text{cn}}}} }}} \right)\cos \theta_{{\text{i}}} } }}{D}$$

It can be seen from Eq. (13) that the proportional coefficient *K* is related to the particle geometric parameter and the normal/tangential strength ratio. During the failure of Brazilian disc specimen, the fractures are substantially in the vertical direction, that is, *θ*_i_ of key contacts is approximately 90° and cos*θ*_i_ is approximately equal to zero. Thus, the influence of cos*θ*_i_ on the scale coefficient *K* can be considered negligible. Numerically, it is shown as much greater sum *m* of vertical projection lengths of key contacts than the sum *n* of horizontal projected lengths. This phenomenon is increasingly evident with the decrease of the minimum ball radius and radius ratio.

14 $$\sigma_{{\text{t}}} = \sigma_{{{\text{cn}}}} \frac{m}{D} = \sigma_{{{\text{cn}}}} K$$

Scale coefficient $K = \frac{m}{D}$ is a dimensionless coefficient, and its physical meaning is: the ratio of vertical projection length sum of key contacts to the diameter of specimen.

Existing studies^22^ indicate that the scale coefficient *K* of the direct tensile specimen exhibits a linear relationship with the particle radius ratio factor $\frac{1}{{1 + {{R_{\max } } \mathord{\left/ {\vphantom {{R_{\max } } {R_{\min } }}} \right. \kern-0pt} {R_{\min } }}}}$, as well as being influenced by both stiffness ratio and geometric characteristic angle. The geometric characteristic angle can be used to describe the micro-geometric features of Particle Flow Code models. For Particle Flow Code models with particles of equal size, the geometric characteristic angle refers to the angle between the line connecting the centers of two contacting particles and the horizontal line. With regards to the aforementioned characteristics, Eq. (14) can be expressed as follows:

15 $$\sigma_{{\text{t}}} = \sigma_{{{\text{cn}}}} \frac{1}{{1 + {{R_{\max } } \mathord{\left/ {\vphantom {{R_{\max } } {R_{\min } }}} \right. \kern-0pt} {R_{\min } }}}}K^{\prime}$$

where *K′* is the scale coefficient only related to the stiffness ratio and the geometric characteristic angle.

## Methods and results

### Choosing microparameters of CBM

To obtain the numerical solution of scale coefficient *K*’, the particle geometric parameters (minimum ball radius, radius ratio) and the normal bond strength are set as the numerical test variables, while the other parameters as constants. The constant parameters are selected based on Fu’s^6^ micro-parameters for slightly weathered sandstone simulation.

The lower limit of particle size range is determined according to the computing power of computer. In this paper, the lower limit is set as 0.1 mm, and the upper limit as 0.4 mm. The range is divided into 7 groups: 0.10, 0.15, 0.2, 0.25,0.3, 0.35 and 0.40 mm. Meanwhile, the value range of radius ratio is divided into 10 groups: 1, 2, 3, 4, 5, 6, 7, 8, 9 and 10. The value range of normal bond strength of particles is divided into 5 groups: 1.0, 5.0, 15.0, 20.0 and 30.0 MPa.

According to the above selection approach, the value ranges of micro-parameters in the numerical test are detailed in Table 7.

**Table 7:** Micro-parameters of Contact Bond Model.

Microparameters (Abbreviation &unit)		Invariant of test	Variables of test
Geometry parameters	Minimum ball radius (*R*_min_)(mm)		7 levels 0.1, 0.15, 0.2,0.25,0.3,0.35,0.4
	Radius ratio (*R*_max_/*R*_min_)		10 levels 1,2,3,4,5,6, 7,8,9,10
Microparameters of Strength	Contact normal bond strength (*σ*_cn_) (Mpa)		5 levels 1.0,5.0,15.0,20.0,30.0
	Contact shear bond strength (*τ*_cs_) (MPa)	50	
	Ball friction coefficient (*μ*)	1.7	
Microparameters of Stiffness	Stiffness ratio (*k*_n_/*k*_s_)	3.5	
	Contact Modulus (*E*_c_)(GPa)	10	

### Numerical modeling based on CBM

The combinations of particle size (7 groups), radius ratio (10 groups) and normal bond strength (5 groups) total 7 * 10 * 5 = 350 groups. The idea of numerical simulation is to observe the effect of normal bond strength variation on the Brazilian strength of under a combination of particle geometric parameters (particle size and radius ratio).

**Figure 10:**
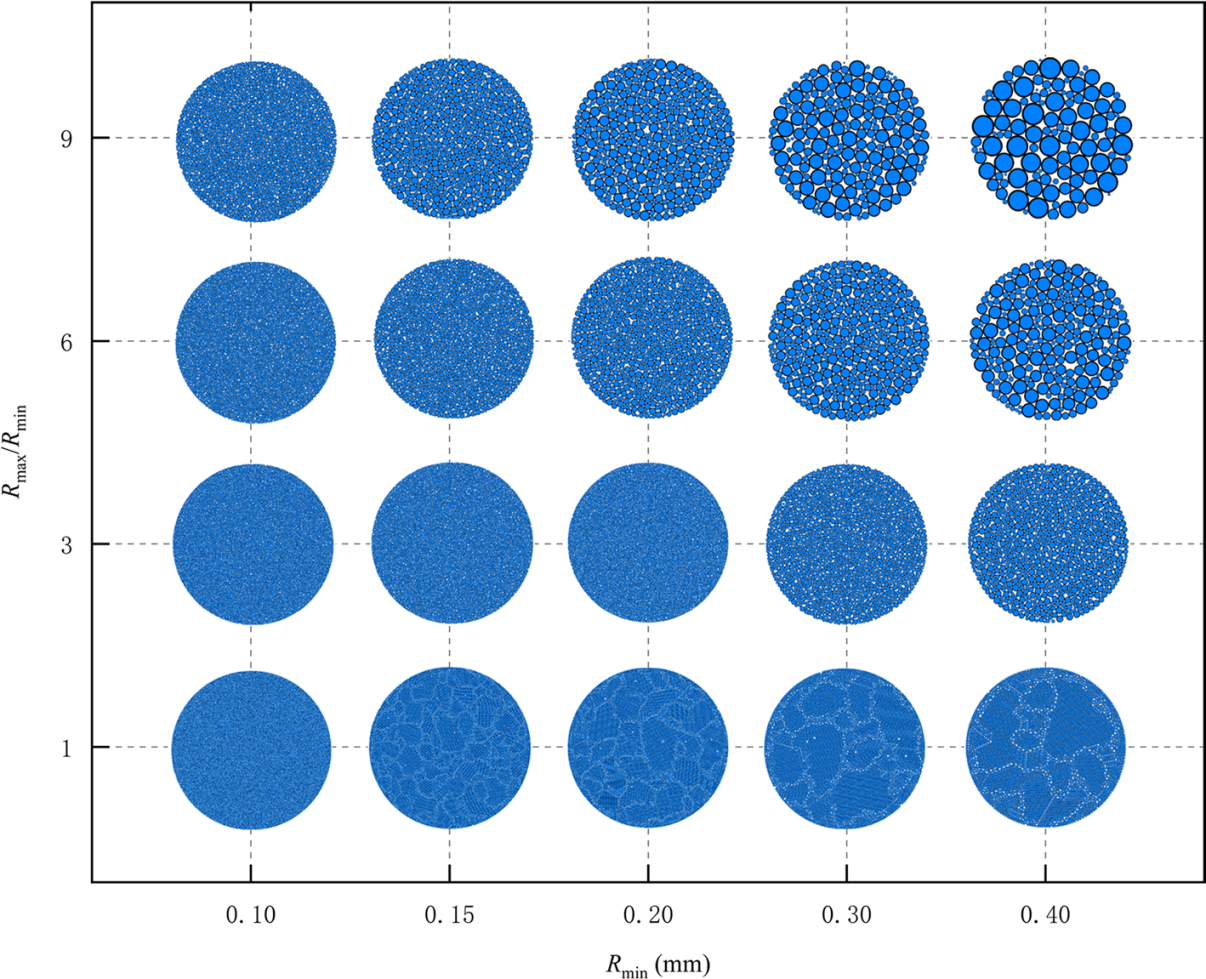
Calculation model of different particle size.

A total of 350 Brazilian numerical test models are built in this paper, and some specimens are shown in Fig. 10. For the specimen with a particle size ratio of 1, the heterogeneity becomes more significant as the particle size increases. This confirms the importance of gradation.

### Effect of Contact Normal Bond Strength on Brazilian strength

**Figure 11:**
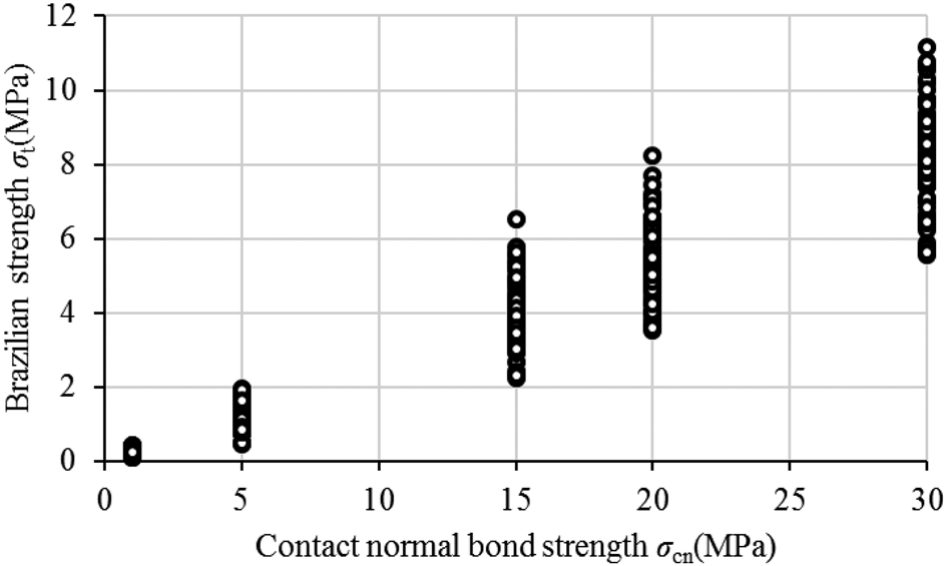
Contact Normal Bond Strength vs Brazilian strength(*R*_max_/*R*_min_ = 1 ~ 8).

Figure 11 shows that the normal bond strength *σ*_cn_ is overall linearly related to the Brazilian strength *σ*_t_. The value range of *σ*_cn_ is 1.0–30.0 MPa (*τ*_cs_ = 50.0 MPa), while the numerically simulated value range of *σ*_t_ is 0.0824–10.705 MPa. It’s worth noting that the specimen will rotate during the loading process when the radius ratio reaches 9 or 10, regardless of the minimum ball radius.

**Figure 12:**
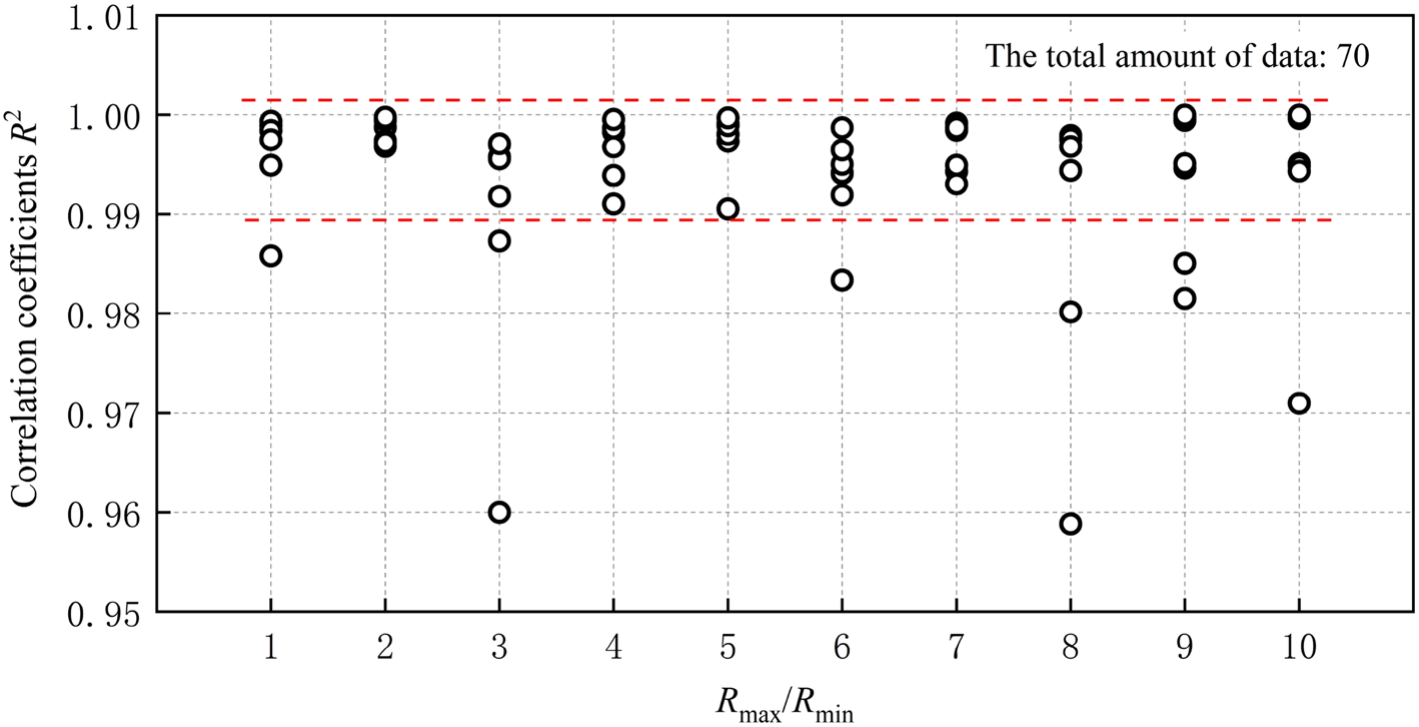
The linear fitting correlation coefficient R2 of different particle size.

Equation (15) is utilized to perform a linear regression analysis (through the origin) on Brazilian tensile strength, normal contact strength, and particle radius ratio factor, in order to determine the scale coefficient *K’*. The slope of the linear regression precisely represents the scale coefficient *K’*. The fitting outcomes and correlation coefficients *R*^2^ are elaborated in Table 8 and Fig. 12.

**Table 8:** Scale coefficient.

*R*_min_ (mm)	*R*_max_/*R*_min_									
	1	2	3	4	5	6	7	8	9	10
0.1	0.6086	0.7677	0.9971	1.0902	1.0862	1.3681	2.0150	2.5678	2.4488	2.1419
0.15	0.5891	0.8609	1.1368	1.2708	1.3031	1.3170	1.6162	2.0441	2.8813	2.8687
0.2	0.6017	0.8169	0.9865	1.2703	1.5834	1.9485	2.5592	1.9833	2.7886	3.4886
0.25	0.7372	0.9698	0.9532	1.2127	1.7335	2.0989	1.9264	1.8702	3.2425	3.7825
0.3	0.6765	0.7791	1.1198	1.3860	1.6116	1.6919	2.1979	2.0543	3.3443	2.4562
0.35	0.6333	0.8389	1.1830	1.3478	1.9879	1.4777	1.8770	2.0077	3.7222	2.3484
0.4	0.5407	1.0640	1.1552	1.2470	1.9475	1.7949	2.1547	2.6982	2.8018	4.3062

The linear regression results for a particle radius ratio of 2 are presented in Fig. 14. The range of *K’* values obtained in this study falls between 0.541 and 4.306. As depicted in Fig. 12, the linear regression analysis yields a correlation coefficient *R*^2^ that predominantly ranges from 0.99 to 1.0. This suggests that within the selected range of microscopic parameters in this study, there exists a strong correlation between the macroscopic tensile strength *σ*_t_ and both the microscopic tensile strength *σ*_cn_ and particle radius ratio factor.

### Effect of particle size on scale coefficient

To further study the variation law of the scale coefficient *K’*, the influence of particle size on *K’* is observed with the particle geometrical parameters (minimum ball radius *R*_min_ and radius ratio *R*_max_/*R*_min_) as the abscissa and the *K′* value as the ordinate.

**Figure 13:**
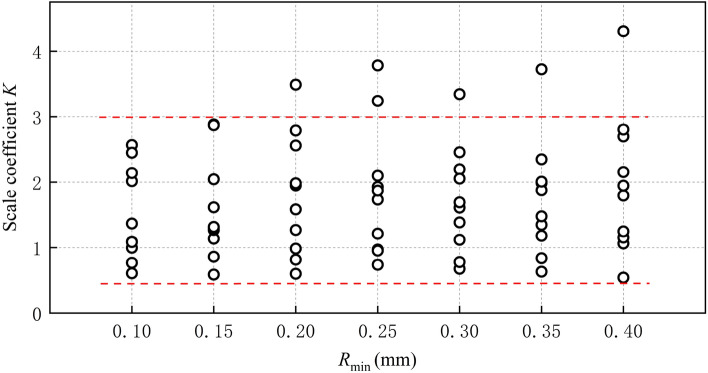
Scale coefficient *K’* vs Minimum ball radius *R*_min_.

**Figure 14:**
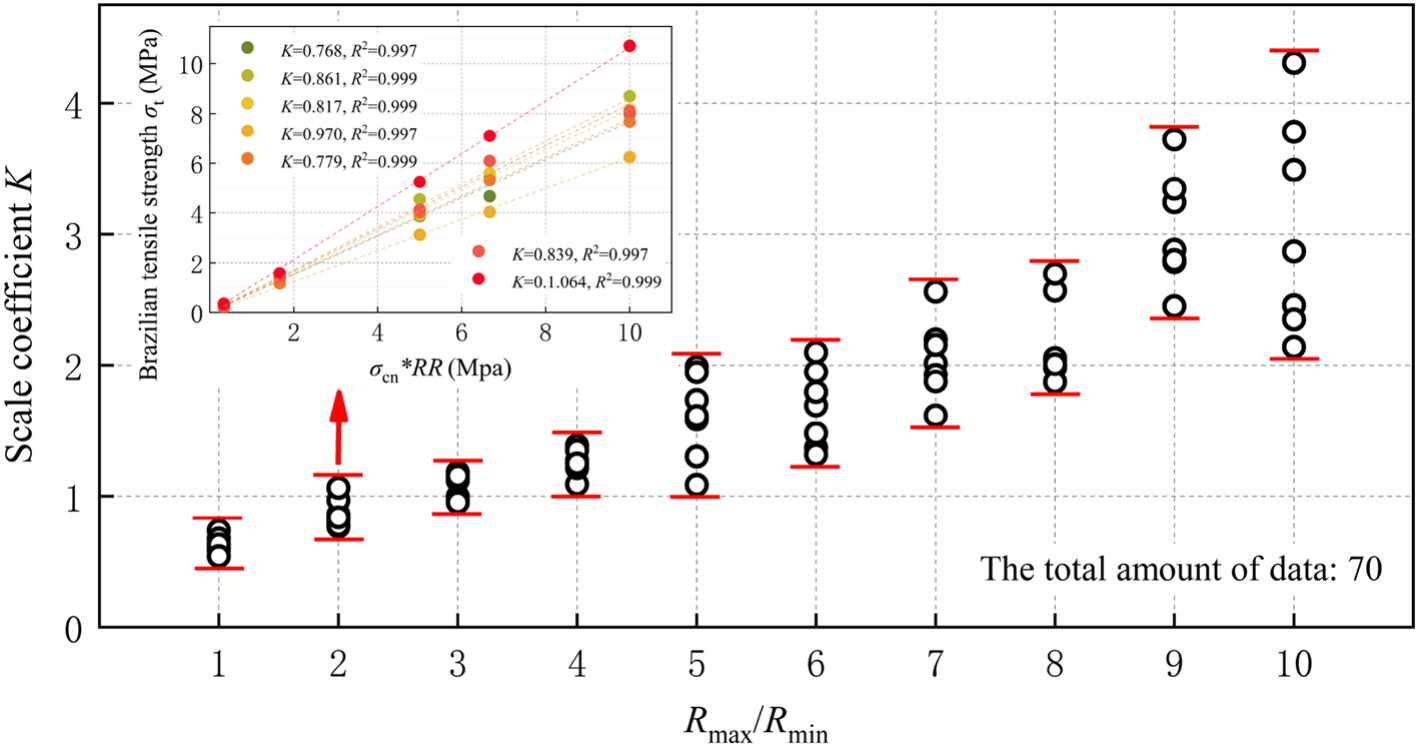
Scale coefficient *K’* vs Radius ratio *R*_max_/*R*_min_.

The minimum ball radius *R*_min_ is not correlated with the scale coefficient *K’*, as illustrated in Fig. 13. As shown in Fig. 14, *K’* still exhibits a strong correlation with *R*_max_/*R*_min_ and demonstrates an increasing trend as the *R*_max_/*R*_min_ increases. This is because the geometric characteristic angle, which is the main influencing factor of *K’*, varies with changes in *R*_max_/*R*_min_. It is noteworthy that the stiffness ratio *k*_n_/*k*_s_ of the specimen in this paper remains constant, thereby implying that *K’* is solely influenced by the geometric characteristic angle. However, the geometric characteristic angle in the specimen cannot be directly measured or calculated. Its influence on the scale coefficient *K’* is complex and therefore does not require calculation during the calibration process of mesoscopic parameters. The correlation between the geometric characteristic angle and the radius ratio will be expounded upon in subsequent investigations with greater detail.

At the same time, we observe that the value of *K′* is more concentrated when *R*_max_/*R*_min_ is between 1 and 4, and more divergent when it is between 5 and 10. This dispersion becomes particularly evident at a *R*_max_/*R*_min_ of 9 or 10. Moreover, the variation pattern between the scale coefficient *K *$\left( {K^{\prime} \times \frac{1}{{1 + {{R_{\max } } \mathord{\left/ {\vphantom {{R_{\max } } {R_{\min } }}} \right. \kern-0pt} {R_{\min } }}}}} \right)$ in this paper and the radius ratio* R*_max_/*R*_min_ is consistent with the pattern of direct tensile testing. Which is when *R*_max_/*R*_min_ is greater than 4, the variation trend of *K* changes.

## Verification

### Steps for calibrating normal bond strength

It can be seen from the above theoretical derivation and Brazil numerical test results that for rock materials with a Brazilian strength between 0.0824 and 10.705 MPa, scale coefficient *K*′ between 0.541 and 4.306, the normal bond strength of particles can be determined according to the following steps:

- The minimum ball radius *R*_min_ and the radius ratio *R*_min_/*R*_max_ are determined based on the microscopic physical composition of rock specimen and computer computing power. According to the computation results of this paper, *R*_min_/*R*_max_ is recommended to be less than 4, whereas the minimum ball radius less than 0.2 mm (Brazilian disc diameter 50 mm).
- At a given Brazilian strength, the normal bond strength of particles is recommended to be estimated by obtaining the scale coefficient *K’* based on the scale coefficient fitting results in Table 8. As shown in the formula:
  $$\sigma_{cn} = \frac{{\sigma_{t} \left( {1 + {{R_{\max } } \mathord{\left/ {\vphantom {{R_{\max } } {R_{\min } }}} \right. \kern-0pt} {R_{\min } }}} \right)}}{{K^{\prime}}}$$
- Recommendations for selection of other micro-parameters are shown in Section “Range of microparameters selection and correction” of this paper (modification procedure for PB design parameter selection).
- Conduct the initial simulation, readjust the the normal bond strength *σ*_cn_ based on the error between the simulated and target values, and perform a subsequent simulation to obtain a more precise calibration value.

### Verification

Sandstone is used as the simulation object in the example verification, whose tensile strength is measured to be 9.08 MPa upon the Brazilian test. According to the steps described in 5.1, six sets of particle micro-parameters are selected separately for verification.

Within the particle size range determined in this paper, the minimum ball radius is selected as 0.07 mm, 0.09 mm, 0.115 mm, 0.135 mm, 0.155 mm and 0.185 mm, while the radius ratio as 3.4, 7.6, 5.8, 1.5, 6.2 and 2.7, respectively. By looking up the Table 8, the scale coefficient *K*_1_’ of initial simulation is taken as 1.1592, 2.0774, 1.9657, 0.8033, 1.746 and 1.099 respectively. After calculation by (15), the normal bond strength can be obtained as 34.46 MPa, 37.59 MPa, 31.41 MPa, 28.26 MPa, 37.44 MPa and 30.57 MPa, respectively. The initial simulation results consist of *σ*_t1_ and *δ*_1_. According to Table 9, there is still some error between σ_t1_ and the target value of σ_t_ = 9.08 MPa, so it is necessary to correct the scale coefficient *K*_1_*'*. By applying the principle that Brazilian tensile strength is directly proportional to the normal bond strength, a more accurate scale coefficient *K*_2_*’* can be calculated using formula $K^{\prime}_{2} = \frac{{\sigma_{t1} \left( {1 + {{R_{\max } } \mathord{\left/ {\vphantom {{R_{\max } } {R_{\min } }}} \right. \kern-0pt} {R_{\min } }}} \right)}}{{\sigma_{cn1} }}$, which are: 1.2474, 2.1529, 2.1974, 0.7419, and 1.2667 respectively. Subsequently, by substituting *K*_2_’, *R*_max_/*R*_min_, and σ_t_ into formula (15), σ_cn2_ can be calculated。 The micro-parameters of six data groups and the numerical simulation results are detailed in Table 9.

**Table 9:** The micro-parameters for numerical simulation.

NO	Microparameters								Macropapraameter and Relative error			
	R_min_ (mm)	R_max_/R_min_	E_c_ (GPa)	k_n_/k_s_	σ_cn1_ (Mpa)	σ_cn2_ (Mpa)	σ_cn_/τ_cs_	μ	σ_t1_ (MPa)	δ_1_(%)	σ_t2_ (MPa)	δ_2_(%)
1	0.07	3.4	10	3.5	34.46	32.03	0.4	1.7	9.77	7.6	9.41	3.63
2	0.09	7.6	16	2.0	37.59	36.27	0.6	1.0	9.41	3.63	8.599	5.3
3	0.115	5.8	12	3.0	31.41	28.1	0.5	1.5	10.15	11.78	9.032	0.53
4	0.135	1.5	8	2.5	28.26	30.6	0.7	1.8	8.386	7.64	8.924	1.72
5	0.155	6.2	15	1.8	37.44	23.91	0.3	2.0	14.22	56.6	8.316	8.414
6	0.185	2.7	13	2.3	30.57	26.52	0.2	2.3	10.466	15.26	9.335	5.12

It can be seen from Table 9 that when both *R*_min_ and *R*_max_/*R*_min_ are large, such as test 5, simulation results tend to be less accurate. The relative error of the initial and secondary simulations for test 5 is 56.6 and 8.414%, respectively. After the secondary simulation, the relative error of other tests is less than 5.3%, which indicates the feasibility of the method for selecting normal bond strength value presented in this paper. The error basically increases with the increase of the mean particle size. The accurate is highter when mean particle size is small. In addition, the other micro-parameters in Table 9 differ in value from Table 8 for the purpose of proving the decisive effect of normal bond strength on Brazilian strength.

## Conclusion

Through the theoretical derivation and numerical simulation of this paper, a method for acquiring the normal bond strength in CBM is established, and the following conclusions are drawn:

1. The factor analysis results of PB design show that the normal bond strength has the most significant effect on Brazilian strength.
2. The linear relationship between the normal bond strength and the Brazilian strength is established through the analytical solution of Brazilian disc and the force analysis of particle model, and the semi-analytical solution of normal bond strength is derived.
3. Good linear correlation between normal bond strength, particle radius ratio factor, and Brazilian strength is verified through 350 runs of numerical simulations. Meanwhile, the correctness of the semi-analytical solution is proved. Fitting and solving of scale coefficient *K'* are done.
4. The fitting results indicate that the scale coefficient *K'* independent of the minimum ball radius, while it is significantly influenced by geometric characteristic angle.
5. The modification procedure of PB design micro-parameters suggests that the particle flow model with simulation results in line with laboratory test results can be obtained when the normal/tangential strength ratio is less than 1, the normal/tangential stiffness ratio is greater than 1, and the ratio of micro-modulus to normal bond strength is within a (0.14–0.83) × 10^3^ range.

## Data Availability

All data included in this study are available upon request through contacting the corresponding author.
